# Chemotherapy plus atezolizumab for a patient with small cell lung cancer undergoing haemodialysis: a case report and review of literature

**DOI:** 10.1002/rcr2.741

**Published:** 2021-03-25

**Authors:** Mihoko Imaji, Daichi Fujimoto, Mai Kato, Masanori Tanaka, Katsuyuki Furuta, Nobuyuki Yamamoto

**Affiliations:** ^1^ Internal Medicine III Wakayama Medical University Wakayama Japan

**Keywords:** Atezolizumab, carboplatin, etoposide, extensive‐stage small cell lung cancer, haemodialysis

## Abstract

Little is known about the safety of chemotherapy plus atezolizumab for patients with extensive‐stage small cell lung cancer (ES‐SCLC) undergoing haemodialysis (HD). An 80‐year‐old male received carboplatin [area under the concentration‐time curve (AUC) = 5 on day 1], etoposide (40 mg/m^2^ on days 1, 2, and 3), and atezolizumab (1200 mg/body on day 1) as the first‐line therapy for ES‐SCLC. He was undergoing HD thrice a week for seven years. HD was provided 16 h after carboplatin administration. During the first cycle, grade 4 neutropenia (neutrophil count: 74/μL) and leukopenia (white blood cell count: 680/μL) occurred. Therefore, chemotherapy was administered with a reduced dose of carboplatin (AUC = 4) and etoposide (30 mg/m^2^) from the second to fourth cycles. After four cycles, no severe non‐haematological adverse events occurred, showing a remarkable response. We conclude that the carboplatin, etoposide, and atezolizumab combination can be safely administered to cancer patients undergoing HD.

## Graphical abstract

We report the clinical course of a patient with extensive‐stage small cell lung cancer (ES‐SCLC) undergoing haemodialysis (HD) who received chemotherapy plus atezolizumab.

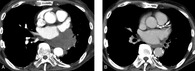

## Introduction

Lung cancer (LC) remains a major cause of cancer incidence and mortality worldwide [1]. Small cell LC (SCLC) accounts for 13–17% of all diagnosed LC cases and is characterized by a rapid proliferation of metastases, high growth fragments, and early development [2]. The prognosis of extensive‐stage SCLC (ES‐SCLC) is poor, with a five‐year survival rate of 6–7% [3].

Platinum‐based chemotherapy has long been the first‐line treatment for ES‐SCLC [4]. However, the development of immune checkpoint inhibitors, such as programmed cell death protein‐1 (PD‐1)/programmed death‐ligand‐1 (PD‐L1) inhibitors, revolutionized the treatment strategy. More recently, the addition of the PD‐L1 inhibitor atezolizumab to the carboplatin and etoposide combination significantly improved overall and progression‐free survival [4]. Therefore, this treatment has become the standard first‐line treatment for ES‐SCLC [4].

Today, >1 million people require long‐term dialysis worldwide [5]. With the increasing number of haemodialysis (HD) patients, physicians are likely to encounter LC patients with chronic renal failure undergoing HD [6]. As patients undergoing HD have limited renal function, they may require dose reduction to avoid overdose and drug toxicity [5]. In addition, drug excretion through HD must be considered for proper chemotherapy timing [5]. The safety of atezolizumab for ES‐SCLC patients undergoing HD has barely been reported.

We report an ES‐SCLC patient undergoing HD who received chemotherapy plus atezolizumab.

## Case Report

An 80‐year‐old male was diagnosed with relapsed stage IV ES‐SCLC (cT4N3M1c; T4: tumour size >7 cm, N3: enlarged lymph nodes in the contralateral mediastinum, and M1c: metastases to the liver, bone, kidney, and retroperitoneum). The patient's performance status was 1. He had been undergoing HD thrice a week for seven years. HD was performed using APS‐21SA (Asahikasei, Japan) for 4 h every session.

We initiated carboplatin (AUC = 5, 125 mg/body on day 1), etoposide (40 mg/m^2^ on days 1, 2, and 3), and atezolizumab (1200 mg/body on day 1) as the first‐line therapy. On day 1 of each cycle, the patient received carboplatin 16 h before dialysis. HD schedule was thrice a week. During the first cycle, thrombocytopenia (grade 1), neutropenia (grade 4, neutrophil count: 74/μL), and leukopenia (grade 4, white blood cell count: 680/μL) were observed. No severe non‐haematological adverse events occurred.

The second, third, and fourth chemotherapy cycles were performed with a reduced dose of carboplatin (AUC = 4, 100 mg/body) and etoposide (30 mg/m^2^), as grade 4 neutropenia occurred during the first cycle. After the initiation of the second cycle, the patient developed grade 3 neutropenia but did not develop any severe non‐haematological adverse events.

**Figure 1:**
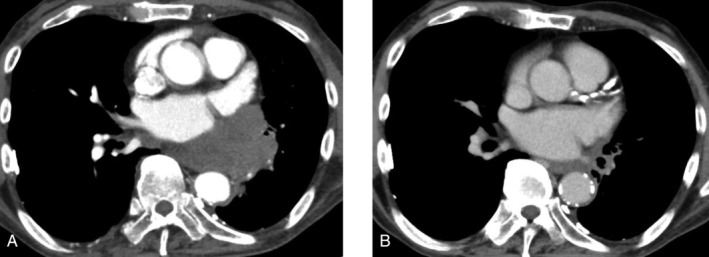
The computed tomography (CT) image before (A) four cycles and after (B) the initiation of this combination therapy. The primary lesion reduced in size.

After four cycles, contrast‐enhanced chest‐abdominal computed tomography (CT) and brain magnetic resonance imaging (MRI) showed marked tumour shrinkage (Fig. 1). At present, the patient has been receiving atezolizumab maintenance therapy for five months.

## Discussion

The patient exhibited a remarkable response to carboplatin, etoposide, and atezolizumab despite the dose and schedule adjustment due to HD. No severe adverse reactions were reported.

The metabolic and excretion routes of each drug in healthy patients are reported as follows: carboplatin is not the first drug to bind into proteins, but the majority of the drugs bind to proteins within 24 h, whereas 55–70% of the drugs are excreted renally within 24 h [7]. Etoposide is excreted renally in 56% of the doses found in urine (45% as an invariant drug), and the remaining 44% is excreted through the bile and stool [8]. Atezolizumab is thought to be metabolized and excreted in the same way as other monoclonal antibodies (mAbs). Because of their molecular size, mAbs are not excreted in the urine but metabolized to peptides and amino acids that can be reused by the body for de novo synthesis of proteins, or are excreted renally [9].

In this case, we administered carboplatin [area under the concentration‐time curve (AUC) = 5, 125 mg/body on day 1] 16 h before dialysis. The culvert formula has been extensively used to determine carboplatin dosing for fixed AUC and glomerular filtration rate (GFR). This formula can be used in patients with end‐stage renal disease undergoing HD by assuming a zero GFR [5, 7]. If HD is provided 12–18 h after infusion, approximately 70% of the carboplatin is removed and the remainder lasts until the next HD. In addition, dialysis 16 h after administration showed the AUC was similar to that of patients with normal renal function [7]. As regards etoposide, dose reduction is recommended in HD patients [5]. The study recommends that the etoposide dose should be reduced by 50% and administered at a dose of 25–75 mg/m^2^/day to avoid haematological toxicity in patients with renal insufficiency. Furthermore, HD does not primarily remove etoposide; hence, it can be administered before or after HD [8]. Finally, given the molecular weight of atezolizumab (145 kDa) and its physicochemical characteristics, drug removal through dialysis is unlikely, with the blood concentration being irrespective of HD [10]. Therefore, we administered carboplatin (AUC = 5, 125 mg/body on day 1), etoposide (40 mg/m^2^ on days 1, 2, and 3), and atezolizumab (1200 mg on day 1). The results of previous reports on PD‐1/PD‐L1 inhibitor for LC patients on HD are summarized in Table 1 [11, 12, 13, 14].

**Table 1:** Published cases who received PD‐1/PD‐L1 inhibitor for lung cancer during HD.

Reference	*N*	Disease	Regimen	Dose (schedule)	HD schedule	Adverse events (grade ≥ 3)	Response
Our case	1	SCLC	CBDCA Etoposide Atezolizumab	125 mg/body (day 1) 40 mg/m^2^ (days 1, 2, and 3) 1200 mg/body (day 1)	16 h after the initiation of CBDCA	Neutropenia, leukopenia	PR
Ishizuka et al. [11]	1	NSCLC	Pembrolizumab	200 mg/body	Detail unknown	None	PR
Chang and Shirai [12]	1	Melanoma	Pembrolizumab	2 mg/kg	Detail unknown	None	CR
Ansari et al. [13]	1	Renal cell carcinoma	Nivolumab	3 mg/kg	Detail unknown	None	PR
Osa et al. [14]	1	NSCLC	Pembrolizumab	200 mg/body	Detail unknown	None	No data

CBDCA, carboplatin; CR, complete response; HD, haemodialysis; NSCLC, non‐SCLC; PD‐1, programmed cell death protein‐1; PD‐L1, programmed death‐ligand‐1; PR, partial response; SCLC, small cell lung cancer.

It is not clear how dialysis affects the blood levels of each drug as the blood concentration was not measured. The patient exhibited a remarkable response and did not develop severe adverse reactions. From Japanese data of the IMpower 133 trial, severe non‐haematological adverse events (grade ≥ 3) occurred in approximately 6% of patients and the response rate was about 75% [15].

In conclusion, we safely administered a combination of carboplatin, etoposide, and atezolizumab to a patient undergoing HD, who demonstrated a remarkable response. This treatment is feasible and effective for SCLC patients undergoing HD.

### Disclosure Statements

Appropriate written informed consent was obtained for publication of this case report and accompanying images.

Dr DF reports personal fees from AstraZeneca KK, Ono Pharmaceutical, Bristol‐Myers Squibb, Taiho Pharmaceutical, Chugai Pharmaceutical, MSD KK, Boehringer Ingelheim Japan, Eli Lilly Japan KK, and Novartis Pharma KK, all outside the submitted work. Dr NY reports grants and personal fees from MSD KK, AstraZeneca, Ono Pharmaceutical, Daiichi Sankyo, Taiho Pharmaceutical, Takeda Pharmaceutical, Chugai Pharmaceutical, Eli Lilly Japan KK, Boehringer‐Ingelheim, Novartis, and Pfizer; personal fees from Thermo Fisher Scientific, Bristol‐Myers Squibb, Life Technologies Japan, Nippon Kayaku, and Merck Biopharma; and grants from Astellas Pharma, Tsumura & Co., Shionogi, AbbVie GK, Amgen, Kyorin Pharmaceutical, Eisai, Terumo, Toppan Printing, and TOSOH, all outside the submitted work. The remaining authors have no conflict of interest.

### Author Contribution Statement

All authors contributed to the study conception. The literature search was performed and the first draft of the manuscript was written by Mihoko Imaji. The manuscript was corrected by Daichi Fujimoto. All authors commented on previous versions of the manuscript. All authors read and approved the final manuscript.
